# Comprehensive characterisation of *Culicoides clastrieri* and *C. festivipennis* (Diptera: Ceratopogonidae) according to morphological and morphometric characters using a multivariate approach and DNA barcode

**DOI:** 10.1038/s41598-020-78053-3

**Published:** 2021-01-13

**Authors:** Leila Hadj-Henni, Zoubir Djerada, Christine Millot, Denis Augot

**Affiliations:** 1 Usc Vecpar-ANSES LSA, EA 7510, SFR Cap Santé, Université de Reims Champagne-Ardenne, 51 rue Cognacq-Jay, 51096 Reims Cedex, France; 2 Department of Medical Pharmacology, EA 3801, SFR CAP Santé, Reims University Hospital, 51, rue Cognacq-Jay, 51095 Reims Cedex, France

**Keywords:** Computational biology and bioinformatics, Ecology, Zoology

## Abstract

Biting midges are widespread around the world and play an essential role in the epidemiology of over 100 veterinary and medical diseases. For taxonomists, it is difficult to correctly identify species because of affinities among cryptic species and species complexes. In this study, species boundaries were examined for *C. clastrieri* and *C. festivipennis* and compared with six other *Culicoides* species*.* The classifiers are partial least squares discriminant analysis (PLS-DA) and sparse partial least squares discriminant analysis (sPLS-DA).The performance of the proposed method was evaluated using four models: (i) geometric morphometrics applied to wings; (ii) morphological wing characters, (iii) "*Full wing*" (landmarks and morphological characters) and (iv)  "*Full model*" (morphological characters—wing, head, abdomen, legs—and wing landmarks). Double cross-validation procedures were used to validate the predictive ability of PLS-DA and sPLS-DA models. The AUC (area under the ROC curve) and the balanced error rate showed that the sPLS-DA model performs better than the PLS-DA model. Our final sPLS-DA analysis on the full wing and full model, with nine and seven components respectively, managed to perfectly classify our specimens. The *C. clastrieri* and *C. festivipennis* sequences, containing both COI and 28S genes, revealed our markers’ weak discrimination power, with an intraspecific and interspecific divergence of 0.4% and 0.1% respectively. Moreover, *C. clastrieri* and *C. festivipennis* are grouped in the same clade. The morphology and wing patterns of *C. clastrieri* and *C. festivipennis* can be used to clearly distinguish them. Our study confirms *C. clastrieri* and *C. festivipennis* as two distinct species. Our results show that caution should be applied when relying solely on DNA barcodes for species identification or discovery.

## Introduction

Biting midges are haematophagous insects that are found in abundance all over the world. They transmit a great number of different pathogens, such as protozoa, filarial worms and more generally speaking many different viruses affecting humans and domestic or wild animals worldwide^1^. In Europe, they are recognised as vectors of the bluetongue virus (BTV) and Schmallenberg (SBV) virus^2,3^ and have had an important impact on the economy and animal welfare ^4–6^.

*Culicoides* is a large and diverse genus which includes approximately 1,340 extant species^7^. The taxonomy of *Culicoides* is almost entirely phenetic (based on overall similarity). The morphological diagnostic characters that are commonly used are often too subtle or difficult to observe to permit reliable species identification (see ^8^, for morphological patterns).Wing pattern is of primary importance in species diagnosis^9^ (^10^ for IIKC) but often shows marked intraspecific variation that can even exceed interspecific variation^11–17^.

There have been attempts to clarify *Culicoides* systematics by using approaches other than traditional morphological diagnostic characters, such as via (i) traditional morphometrics; (ii) geometric morphometrics (GM); (iii) nuclear, mitochondrial and ribosomal DNA analyses and (iv) matrix-assisted laser desorption/ionization-time of flight (MALDI-TOF)^7^. Recent taxonomic revisions based on these alternative characters (morphology characters, molecular and/or morphometric tools) have led to the description of new species within the genus^18–23^. The rise of DNA barcoding and lack of taxonomic experts conducted Ander^24^ to propose COI sequencing as a tool for rapid identification of *Culicoides* species. Finally, recent contributions to *Culicoides* taxonomy at the species level have revealed a close correlation between morphological and molecular analyses^7^.

Most of the studies on insects of medical, veterinary or economic importance use the landmark approach for quantitative assessment and visualisation of morphological variations within and among species. As such, wings have been the subject of many geometric morphometric analyses in insects^25^. In *Culicoides,* it was shown that wing shape variation can discriminate species and cryptic species^26–30^.

A current trend known as integrative taxonomy has recently been applied to delineate species where traditional pattern-based taxonomy failed to accurately find their limits, such as species complexes and groups or cryptic species. Large-scale integrative taxonomic efforts incorporating morphological, ecological, and independent multi-locus sequence data from species sampled across their known ranges provide the best means to test species boundaries and refine essential species distribution data^31^.

Our study uses standardised samples to assess diagnostic characters within two closely-related species: *C. clastrieri* Callot, Kremer & Deduit 1962 and *C. festivipennis*, Kieffer 1914. We focus on different kinds of characters, from (i) morphology; (ii) GM; (iii) mitochondrial DNA; and (iv) ribosomal DNA.

According to Sarvasova^22^, *C. festivipennis* can be distinguished from *C. clastrieri* by *sensillae coeloconia* (number and distribution). The DNA barcode is unable to distinguish *C. clastrieri* from *C. festivipennis*^22, 24,32^. The DNA divergence, based on COI Genbank sequences, is wider in *C. festivipennis* specimens than between *C. festivipennis* and *C. clastrieri*: 1.2% and 0.7% respectively (Augot, personal communication).

In our integrative approach to taxonomy, *C. alazanicus* Dzhafarov, 1961, *C. brunnicans* Edwards, 1939, *C. circumscriptus* Kieffer, 1918, *C. furcillatus* Callot, Kremer and Paradis, 1962, *C. pictipennis* (Staeger), 1839 and C. *nubeculosus* (Meigen), 1830 were added to the analyses for molecular and taxonomic reasons^22,24^ and because the species were available in our lab.

In our study, we applied the classification performance of the partial least squares discriminant analysis (PLS-DA) and sparse partial least squares discriminant analysis (sPLS-DA) models, widely-used classifiers. The specific objective was to investigate the classification performance of PLS-DA and sPLS-DA to find a satisfactory combination of morphometric and morphological datasets. ROC (receiver operating characteristic) curves were used to assess and optimise the specificity and sensitivity of each class with different thresholds.

## Results

### Molecular analyses

#### Results of molecular analyses

The sequences obtained are available in GenBank (Supplementary Information 1). Sequence alignments were 399 bp for COI and 587 bp for 28S including gaps.

### Phylogenetic analysis

**Figure 1:**
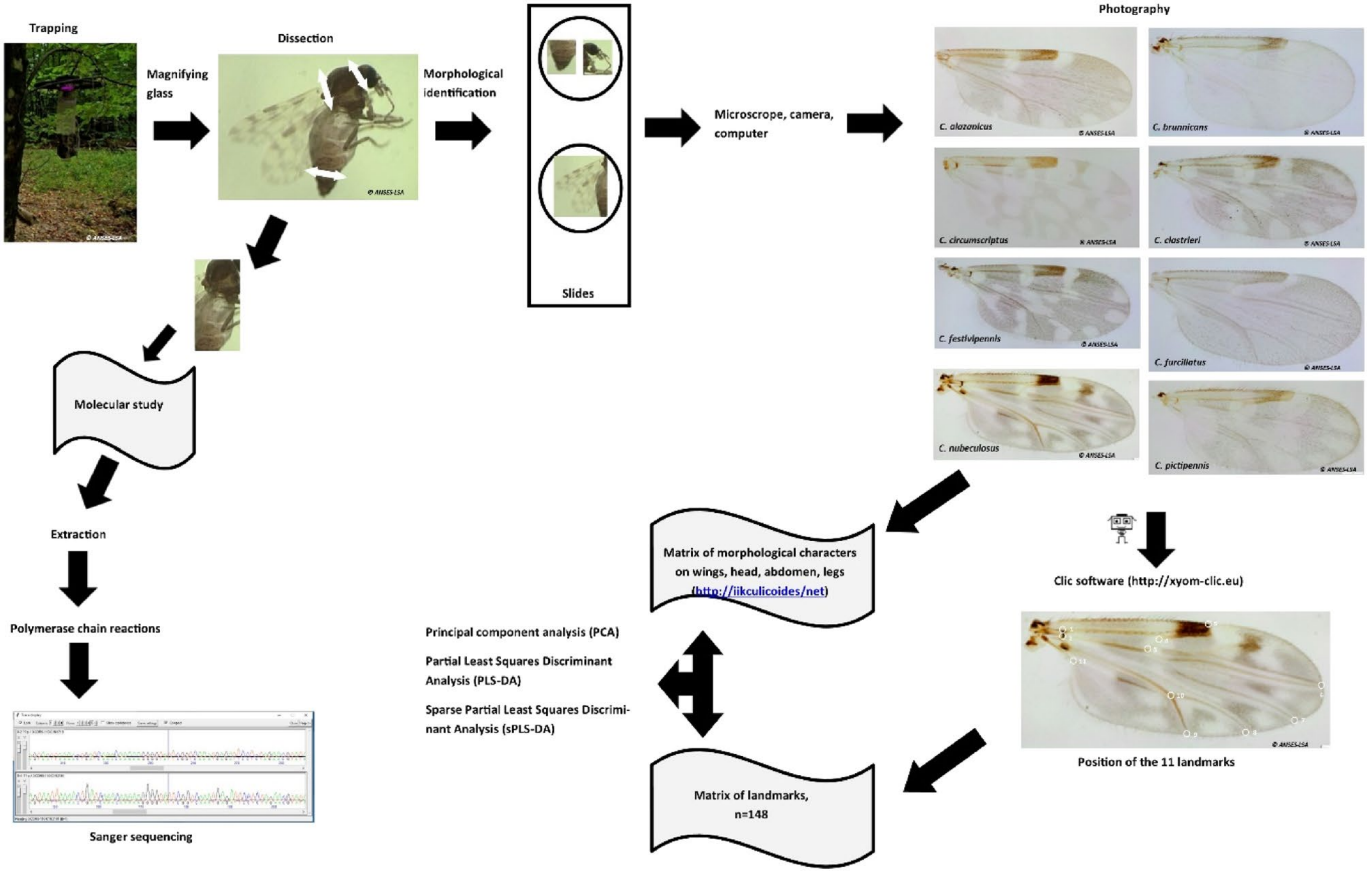
Block diagram of the study.

Our molecular analysis (Fig. 1) with both markers generated seven supported clusters, six of which were in agreement with the morphological determination (i.e. *C. alazanicus, C. brunnicans*, *C. circumscriptus*, *C. furcillatus, C. nubeculous* and *C. pictipennis*). However, one cluster (i.e. two species) corresponded to undistinguished *C. clastrieri* and *C. festivipennis.*

In addition, the COI mtDNA tree shows that *C. furcillatus* is the sister of the “*C. clastrieri*/*festivipennis*” clade. Indeed, *C. pictipennis* is the sister species of *C. brunnicans* while *C. circumscriptus* is positioned between the two clades.

Moreover, the 28S rDNA tree shows that *C. pictipennis* is the sister of the “*C. clastrieri*/*festivipennis*” clade. The other species are positioned in several places without a clade.

### Intra- and inter-specific comparison

The COI Genbank sequences show little intraspecific divergence in both *C. clastrieri* (0.1 ± 0.1%) and *C. festivipennis* (1.2 ± 0.4%). The interspecific difference between *C. clastrieri* and in *C. festivipennis* is 0.7 ± 0.2%.

Small intraspecific divergences with COI sequences were observed in our sample: *C. alazanicus* (1.2 ± 0.4%), *C. brunnicans* (0.7 ± 0.2%), *C. circumscriptus* (2.2 ± 0.5%), *C. clastrieri* (0.3 ± 0.1%), *C. festivipennis* (0.4 ± 0.1%), *C. furcillatus* (1.5 ± 0.4%), *C. nubeculosus* (0.2 ± 0.1%) and *C. pictipennis* (1.1 ± 0.3%).

Finally, C*. festivipennis* and *C. clastrieri*—grouped in the same main clade—showed small interspecific distances (0.4 ± 0.2%); these were not identified as separate species based on DNA barcodes. We therefore decided to create a new group (*C. clastrieri/festivipennis* clade) based on interspecific distance. The overall mean genetic distance (K2P) computed for the different species of *Culicoides* was found to be 16.6 ± 1.4%. Interspecific K2P values for different (Table 1) species and taxa ranged from 27.3% (between *C. furcillatus* and *C. nubeculosus;* between *C. circumscriptus-*and *C. furcillatus*) to 17.2 ± 2.1% (between *C. circumscriptus* and the *C. clastrieri/festivipennis* clade) for our samples. For the COI Genbank sequences, we observed approximatively the same proportion and the same species (Table 1). We remarked very little interspecific divergence between our sample of the C. *clastrieri*/*festivipennis* clade and the C. *clastrieri*/*festivipennis* Genbank clade (0.6 ± 0.4%).

**Table 1:** Estimation of pairwise distance (± SD) of the *Culicoides* species for the COI domain of the mtDNA and D1D2 region of the rDNA.

	Species	COI sequences													
		1	2	3	4	5	6	7	8	9	10	11	12	13	14
1	*C. alazanicus*-Genbank		0.023	0.024	0.024	0.028	0.024	0.023	0.004	0.023	0.024	0.024	0.027	0.024	0.023
2	*C. brunnicans*-Genbank	0.181		0.025	0.024	0.029	0.024	0.021	0.023	0.002	0.025	0.024	0.028	0.024	0.023
3	*C. circumscriptus*-Genbank	0.199	0.199		0.021	0.030	0.023	0.020	0.024	0.025	0.006	0.021	0.030	0.023	0.022
4	*C. clastrieri*-festivipennis-Genbank	0.194	0.195	0.167		0.027	0.025	0.022	0.024	0.024	0.021	0.003	0.026	0.025	0.023
5	*C. furcillatus*-Genbank	0.234	0.235	0.248	0.221		0.030	0.027	0.028	0.028	0.030	0.027	0.005	0.030	0.028
6	*C. nubeculosus* –Genbank	0.189	0.187	0.182	0.211	0.252		0.027	0.024	0.024	0.023	0.025	0.030	0.004	0.028
7	*C. pictipennis*-Genbank	0.187	0.158	0.169	0.183	0.231	0.224		0.023	0.021	0.020	0.022	0.026	0.027	0.010
8	*C. alazanicus*	**0.014**	0.181	0.198	0.193	0.235	0.186	0.183		0.023	0.024	0.024	0.027	0.024	0.023
9	*C. brunnicans*	0.180	**0.006**	0.203	0.197	0.234	0.188	0.161	0.180		0.025	0.024	0.028	0.024	0.023
10	C. *circumscriptus*	0.202	0.201	**0.024**	0.174	0.258	0.185	0.178	0.200	0.204		0.021	0.030	0.023	0.022
11	*C. clastrieri/C. festivipennis*	0.192	0.193	0.165	**0.007**	0.221	0.209	0.182	0.191	0.195	0.172		0.026	0.025	0.023
12	*C. furcillatus*	0.248	0.253	0.273	0.236	**0.030**	0.273	0.246	0.248	0.251	0.273	0.235		0.030	0.027
13	*C. nubeculosus*	0.190	0.187	0.186	0.213	0.252	**0.006**	0.226	0.187	0.188	0.190	0.209	0.273		0.028
14	*C. pictipennis*	0.183	0.171	0.177	0.180	0.235	0.229	**0.058**	0.180	0.173	0.186	0.177	0.247	0.230	

		D1D2 sequences													
		1	2	3	4	5	6	7							
1	*C. alazanicus*		0.006	0.006	0.006	0.006	0.009	0.007							
2	*C. brunnicans*	0.025		0.005	0.005	0.004	0.009	0.006							
3	*C. circumscriptus*	0.025	0.016		0.005	0.004	0.009	0.005							
4	*C. clastrieri/C. festivipennis*	0.025	0.018	0.016		0.004	0.009	0.004							
5	*C. furcillatus*	0.021	0.012	0.012	0.012		0.009	0.005							
6	*C. nubeculosus*	0.051	0.047	0.053	0.051	0.051		0.009							
7	*C. pictipennis*	0.027	0.019	0.018	0.013	0.016	0.051								

Analysis from 28S rDNA sequences did not show any intraspecific divergence whatever the taxa (0.000) with the exception of *C. nubeculosus* (0.1 ± 0.1%) and *C. festivipennis/C.clastrieri* (0.1 ± 0%). The overall mean genetic distance (K2P) computed for the different species of *Culicoides* was found to be 2.1 ± 0.03%. Interspecific K2P values for different species (Table 1) and taxa ranged from 1.2% (between *C. circumscriptus* and *C. furcillatus*; *C. furcillatus* and *C. brunnicans*, the main C. *clastrieri*/*festivipennis* clade and *C. furcillatus)* to 5.3 ± 0.9% (between *C. circumscriptus* and *C. nubeculosus*).

### Morphometric and morphological analyses

**Figure 2:**
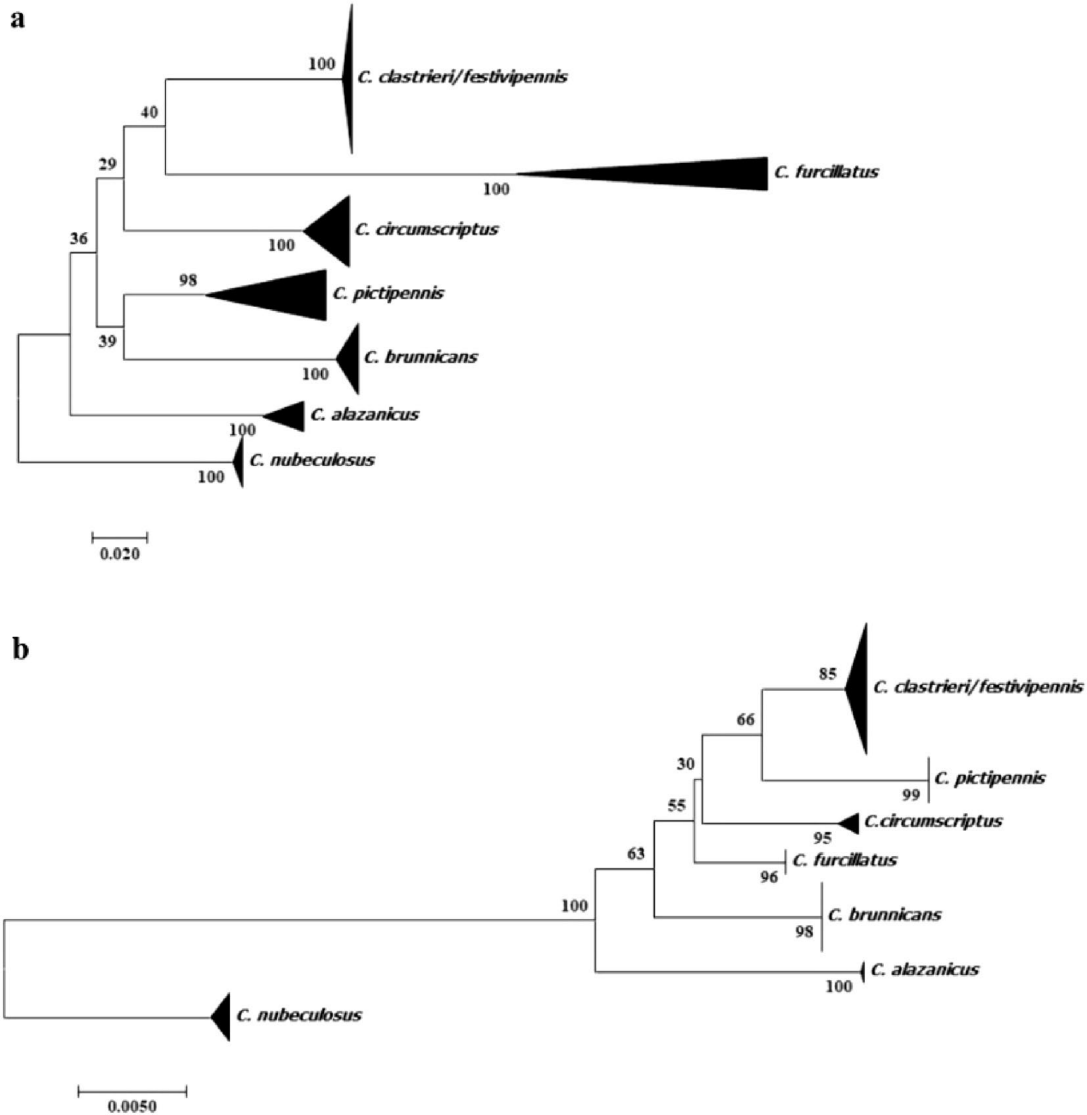
Trees obtained from nucleotide analysis of: (**a**) COI mtDNA; (**b**) 28S rDNA (with MP method) sequences of *C. alazanicus, C. brunnicans, C. circumscriptus C. clastrieri, C. festivipennis, C. furcillatus, C. nubeculosus and C. pictipennis* and bootstrap values are shown in nodes (1000 replicates).

In all, 148 specimens identified as *C. alazanicus* (n = 10), *C. brunnicans* (n = 27), *C. circumscriptus* (n = 27), *C. clastrieri* (n = 21), *C. festivipennis* (n = 20), *C. furcillatus* (n = 14), *C. nubeculosus* (n = 19) and *C. pictipennis* (n = 20) were analysed with 11 wing landmarks/specimens (Fig. 2).

### Principal component analyses

Principal component analysis (PCA) was used to observe possible grouping trends.

**Figure 3:**
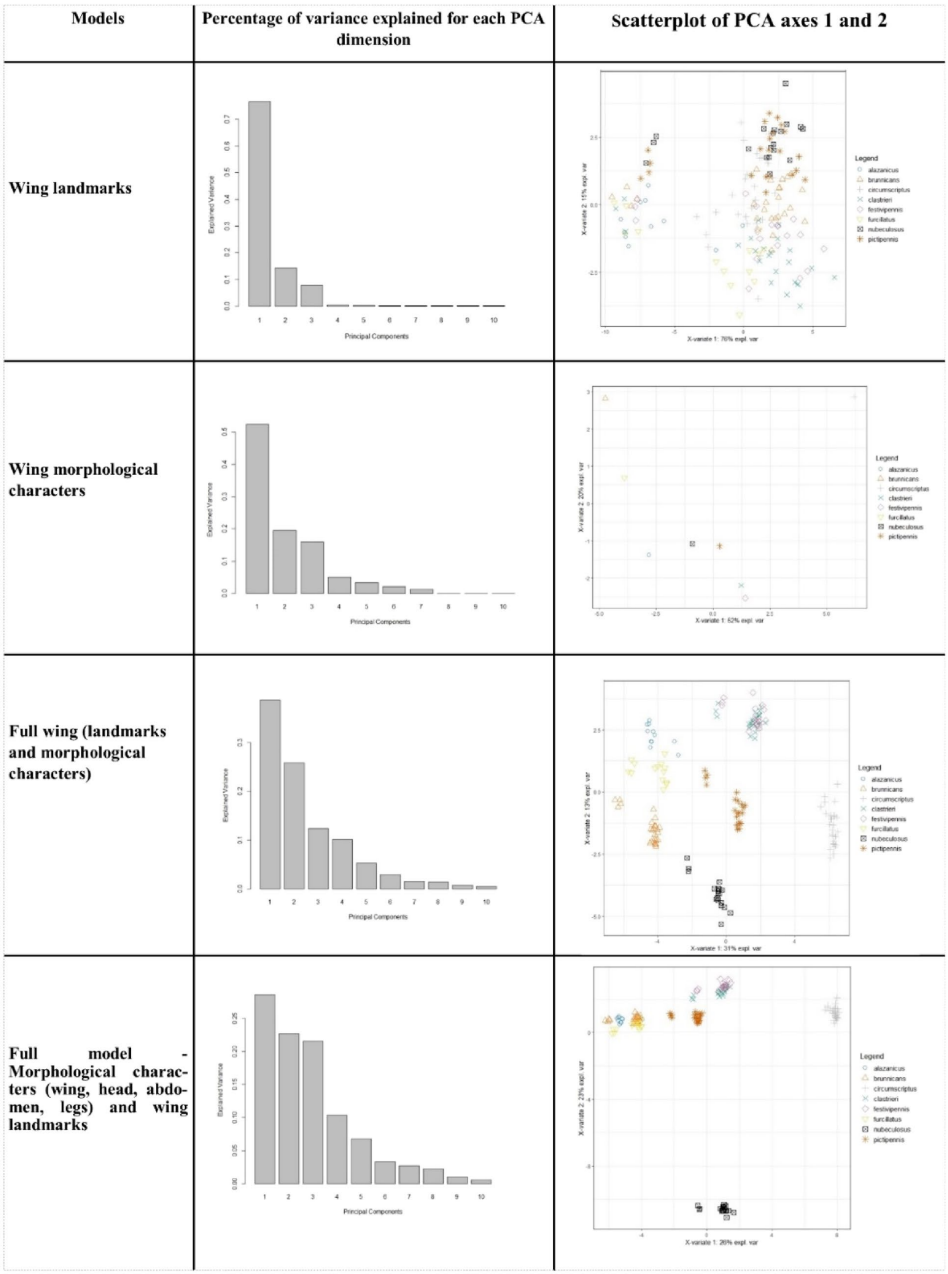
Principal component analysis (PCA): percentage of variance explained for each PCA dimension and results.

Firstly, we performed a first normed PCA using the “*Wing landmarks*” model. The first three axes accounted for 76%, 15% and 8% of the total variance, which suggests a weak structuration of the data. This was confirmed by a scatterplot of PCA axes 1 and 2 that was unable to separate the species (Fig. 3).

Secondly, we performed a first normed PCA on the “*Wing morphological characters*” model. The various specimens of each species are represented by a single point suggesting a close correlation of wing morphological characters. This model, without variance, is not validated and does not permit species separation.

We studied the “*Full wing* (landmarks and morphological, characters)” model through a normed PCA on raw data. *C*. *clastrieri* could be clearly separated from *C. festivipennis*. The first five axes accounted for 40%, 25%, 12%, 10% and 5% of the total variance. The scatterplot separated unambiguously and without overlap *C. clastrieri-C. festivipennis* on the one hand and the six species on the other hand (Fig. 3).

Finally, we performed a first normed PCA on the “*Full model*” (Morphological characters—wing, head, abdomen, legs—and wing landmarks). The first nine axes accounted for 26%, 23%, 22%, 10%, 8%., 4%, 3%, 2% and 1% of the total variance, which reveals good structuration of the data. This was confirmed by a scatterplot of PCA axes 1 and 2 that presents the same topology as the wing morphological model (Fig. 3).

This supports discrimination according to the species’ wing pattern. Similarly, and some body pattern characters could be used to identify *Culicoides* from the *clastrieri/festivipennis* clade better and quicker. With that objective in mind, we performed analyses on three datasets: (1) “*Wing landmarks*” (11 landmarks); (2) “*Full wing*” (38 items) and (3) the “*Full model*” that includes 71 items.

### Discriminant analyses

**Figure 4:**
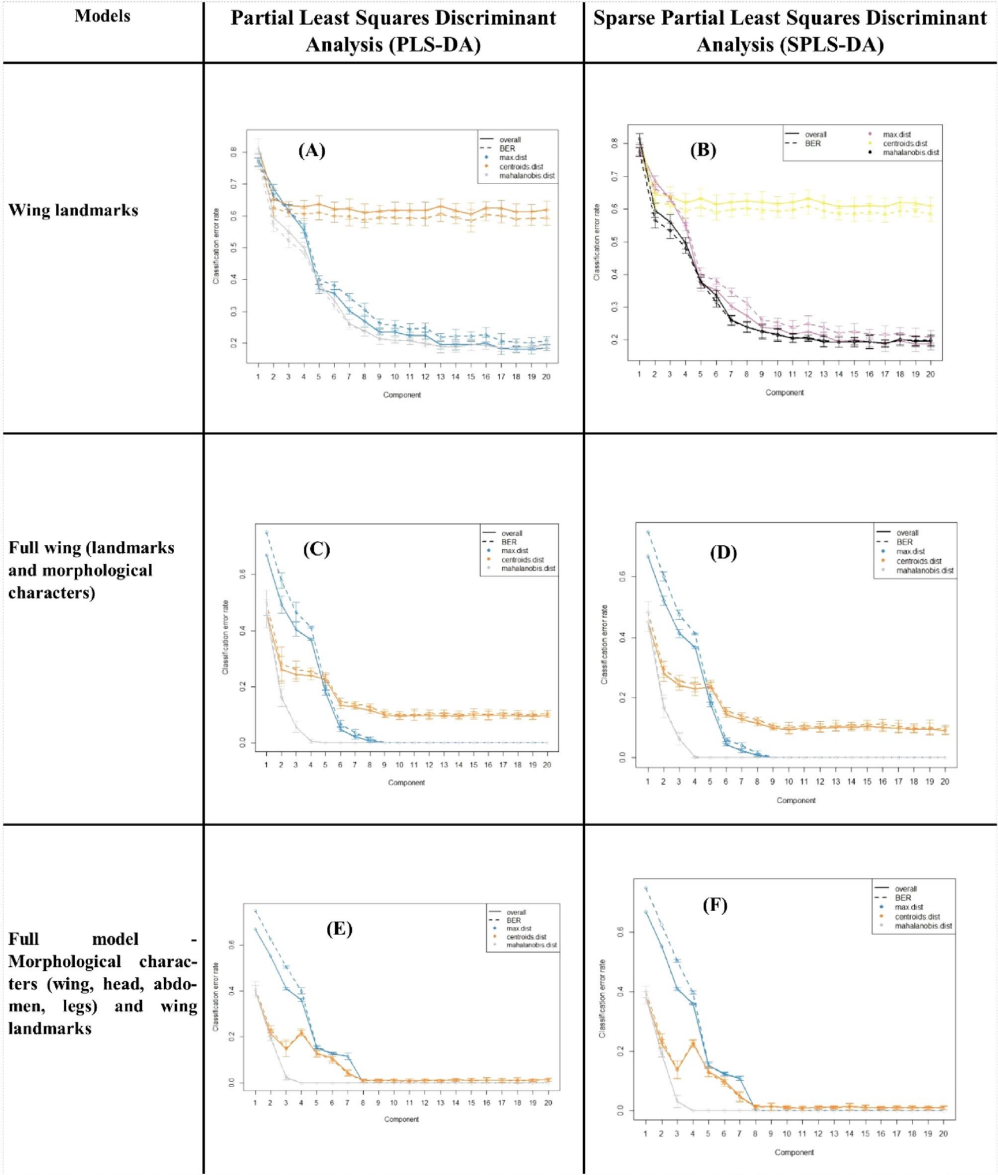
Balanced error rate (BER) choosing the number of dimensions. Performance and ncomp selection.

PLS-DA and sPLS-DA models were used in order to discriminate the extremes (i.e. the most sensitive and most robust groups) using the three datasets (species, models and components) as described. The accuracy and the balanced error rate (BER) for the two models were compared and are summarised in Supplementary Information 2 and Fig. 4.

The tuning step of the number of components to select showed that 16 components were necessary to lower the BER (Fig. 4A,B) for the “*Wing landmarks*” data. The AUC values with 16 components are as follows: *C. alazanicus* (0.97, p < 0.001), *C. brunnicans* (0.98, p < 0.001), *C. circumscriptus* (1.00, p < 0.001), *C. clastrieri* (0.97, p < 0.001), *C. festivipennis* (0.89, p < 0.001), *C. furcillatus* (0.97, p < 0.001), *C. nubeculosus* (1.00, p < 0.001) and *C. pictipennis* (1.00, p < 0.001). After 16 components, the AUC values are approximately comparable (Fig. 4).

From the performance plot (Fig. 4), we observe that the overall error rate and the BER are similar for the “*Full wing*” model (Fig. 4C,D) and the full model (Fig. 4E,F), and decrease when components increase from one to eight. The error rates stabilise after nine components for PLS-DA and sPLS-DA models for “*Full wing*” (Fig. 4C,D). The AUC values with nine components (Supplementary Information 2) are as follows: *C. alazanicus* (1.00, p < 0.001), *C. brunnicans* (1.00, p < 0.001), *C. circumscriptus* (1.00, p < 0.001), *C. clastrieri* (1.00, p < 0.001), *C. festivipennis* (1.00, p < 0.001), *C. furcillatus* (1.00, p < 0.001), *C. nubeculosus* (1.00, p < 0.001) and *C. pictipennis* (1.00, p < 0.001).

In contrast, the error rates stabilise after eight components for PLS-DA and sPLS-DA for the full model (Fig. 4E,F). The AUC values with eight components (Supplementary Information 2) are as follows: *C. alazanicus* (1.00, p < 0.001), *C. brunnicans* (1.00, p < 0.001), *C. circumscriptus* (1.00, p < 0.001), *C. clastrieri* (1.00, p < 0.001), *C. festivipennis* (1.00, p < 0.001), *C. furcillatus* (1.00, p < 0.001), *C. nubeculosus* (1.00, p < 0.001) and *C. pictipennis* (1.00, p < 0.001).

**Figure 5:**
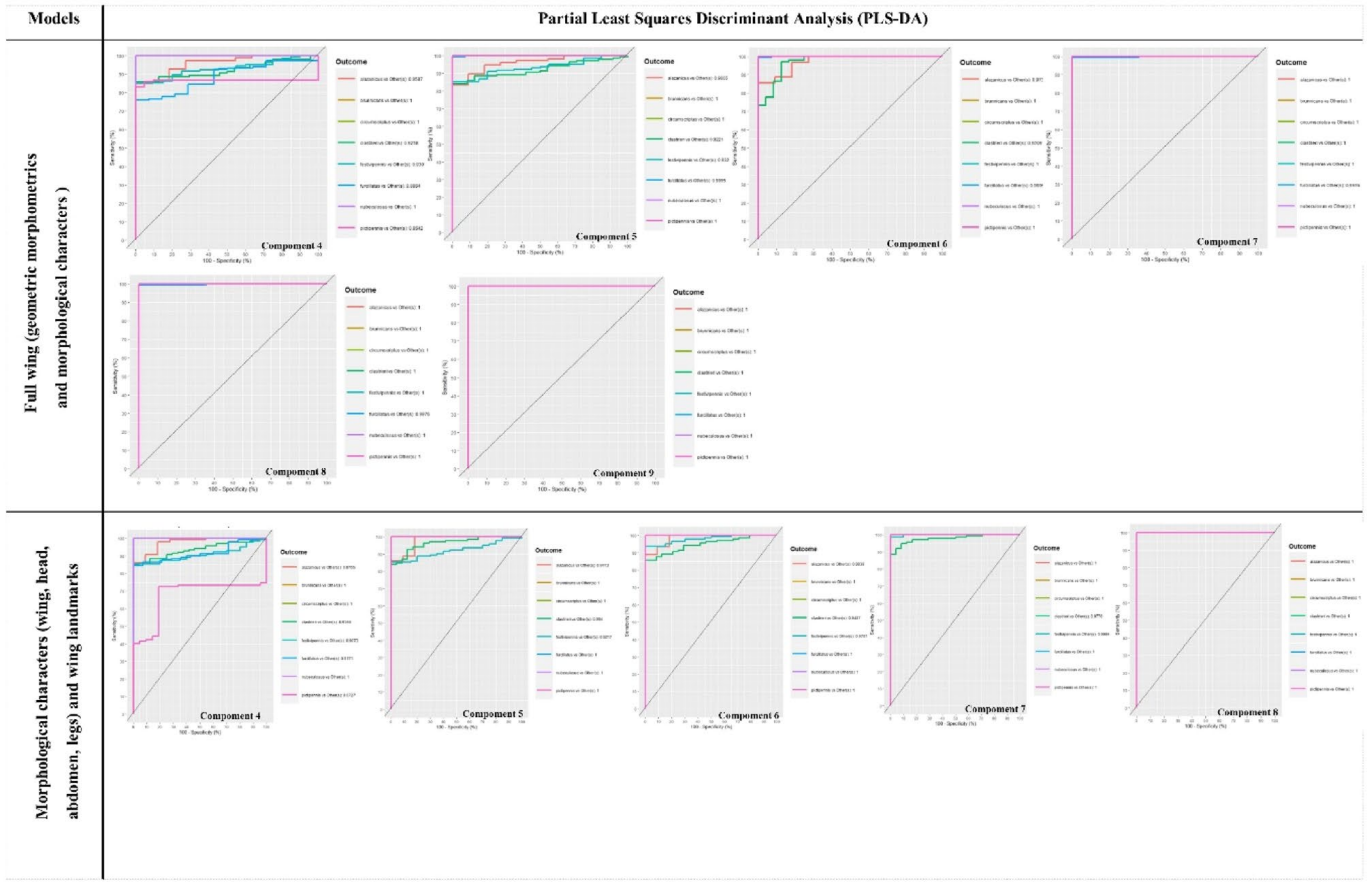
The ROC curve of the “*Full wing*” and “*Full model*” obtained with partial least squares discriminant analysis (PLS-DA) according to the components. A perfect result would be an area under the curve (AUC) of 1.0.

**Figure 6:**
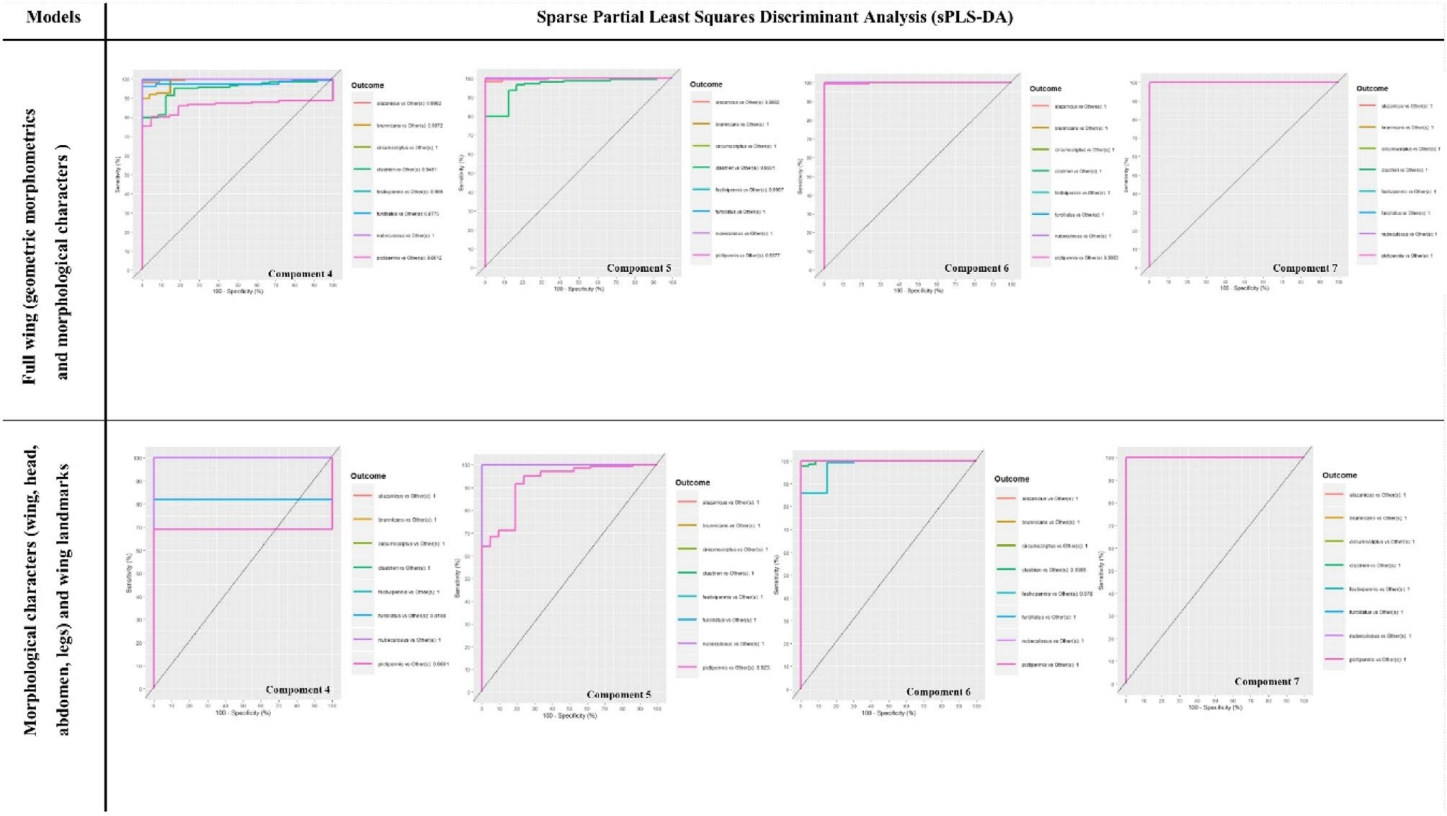
The ROC curve of the “*Full wing*” and “*Full model*” obtained with sparse partial least squares discriminant analysis (sPLS-DA) according to the components. A perfect result would be an area under the curve (AUC) of 1.0.

A perfect result would be an AUC of 1.0 obtained using eight components with PLS-DA (Fig. 5) and seven with sPLS-DA using the “*Full model*” (Fig. 6). For the “*Full wing*” model, nine components with PLS-DA (Fig. 5) and seven with sPLS-DA (Fig. 6) are needed to obtain an AUC of 1.0.

The most discriminating characters for the "*Full wing*" and "*Full model*" with PLS-DA and sPLS-DA are summarised in Supplementary Information 3 and 4 respectively.

With the PLS-DA classifier and the “*Full model*”, we observe that a large number of characters are necessary to separate species. According to the components, 39 items were identified for *C. clastrieri* (× 11, × 2, × 1, WingM18, × 4, y2, AnM54, y11, Palp.M51, y9, × 9, × 2, Wing.M11, y2, y5, y11, y6, Ab.M30, Ab.M29, y1, y4, y5, Wing.M8, Ant.M54, y3, Palp.M50, Wing.M2, Ab.M28, × 1, y10, Ant.M52, Wing.M7, Wing.M8, , Wing.M13, × 7, Phr.M48, y8, × 11, Ab.M30) and eight for *C. festivipennis* (Wing.M12, Ant.M52, WingM12, WingM4, y11, Ant.M58, Ant.M54, WingM11). For example, for *C. pictipennis*, seven items (y2, × 5, y10, y3, × 8, Wing.M4, × 6) were observed. For the other species, see details in Supplementary Information 3. Fewer descriptors are needed for species discrimination with the sPLS-DA model than with the PLS-DA model. Fourteen descriptors were needed for *C. clastrieri* (y3, × 8, Wing.M2, y4, Wing .M9, y5, y1, y8, Palp.M51, × 4, × 11, Wing.M20, Ant.M56, Wing.M12) and one for *C. festivipennis,* (Wing.M12). Only two items are needed to identify *C. pictipennis* (Ant.M57, Wing.M25) and three for *C. furcillatus* (Ab.M33, Ant.M58, Ab.M29). For the other species, see details in Supplementary Information 4.

With the PLS-DA classifier and the “*Full wing*”, we observe that many items are necessary to discriminate species. According to the components, 31 items were identified for *C. clastrieri* (× 1, × 2, × 3, × 4, × 6, × 9, × 10, × 11, y1, y2, y3, y4, y5, y6, y7, y9, y11, WinM.2, WingM.3, WingM.6, WingM.7, Wing.M8, Wing.M9, Wing.M10, Wing.M11, Wing.M13, Wing.M14, WingM.15, WingM.16, Wing.M18, Wing.M19) and eight for for *C. festivipennis* (× 1, × 7, y10, Wing.M1, WingM.10, Wing.M11, Wing.M12, Wing.13). For the other species, see details in Supplemental Data S3. In contrast, with the sPLA-DA classifier, only a few descriptors are needed for species classification. Five descriptors were needed for *C. clastrieri* (y1, y2, y3, y4, y5) and one for *C. festivipennis,* (Wing.M12). For the other species, see details in Supplementary Information 4.

## Discussion

The present integrative taxonomy study carried out on two closely-related and sympatric species, *C. clastrieri* and C*. festivipennis,* shows congruence between classical morphological identification and GM results. The molecular data revealed a joint C*. clastrieri/festivipennis* clade.

This paper reports a comprehensive evaluation of selected statistical classification techniques (PLS-DA and sPLS-DA) to discriminate species on the basis of four models: (i) “GM wings”; (ii) “morphological wing characters”, (iii) “*Full wing*” model and (iv) “*Full model*”. While these classifiers have been used in several scientific domains (particularly medicine) their performance had never previously been assessed on morphological and GM data for insects. sPLS-DA is clearly competitive in terms of computational efficiency and superior in terms of interpretability of results; it is therefore a good alternative to other types of discriminant models ^33^.

The application of a GM approach confirmed the separation of species with AUC values of 0.91 (*C. festivipennis*), 0.98 (*C. alazanicus*, *C. clastrieri* and *C. furcillatus*), 0.99 (*C. brunnicans*) and 1.0 (*C. circumscriptus*, *C. nubeculosus* and *C. pictipennis*), indicating that this technique is a powerful tool for discriminating closely-related species^26–30^ and could possibly be used to separate species as yet considered cryptic. The GM analysis scores show correct identification in female specimens of 77.8% to 100%^26–30^.

It is interesting to note that the sPLS-DA classifier was able to separate species in the “*Full wing*” model and the “*Full model*” with AUC values of 1.0. The character classifier based on 14 items performed better than that based on 39 items for *C. clastrieri* with the full model. For *C. festivipennis*, just one item is necessary to discriminate this species. For  the “*Full wing*” model, the sPLS-DA classifier can characterise *C. clastrieri * with five items (landmarks) while only one item (morphological character) is needed for *C. festivipennis*.

Our study, combining both GM and morphological characters, allows very good discrimination of our specimens. The classifiers were tailored so as to reduce the number of items needed to characterise species.

The "*Full wing*” (landmarks and morphological, characters) model separates the species without error. The dichotomous keys to species include all morphological characters and are used to identify *Culicoides* fauna by biogeographical region^7^. Moreover, identification aids based on wing patterns have been published for the same regions^7^. To our knowledge, only one study based on wing patterns has produced identification keys for epidemiological studies^9^. However, the final identification is the species or species group. Our study, based on both the morphological characters of wings (27 items) and the application of GM (11 points), allows all the studied species to be successfully separated, including cryptic species (*C. clastrieri* and *C. festivipennis*). Our study relies on the standardisation of morphological characters (http://www.iikculicoides.net). Future investigations are needed to create a worldwide database and to combine the GM approach for identifying *Culicoides* species.

DNA barcoding based on the COI and 28S sequences discriminated all morphologically determined species except *C. festivipennis* and *C. clastrieri,* which are not considered as separate species using these analyses. The identical *C. festivipennis/clastrieri* sequences with both COI and 28S, the rDNA gene assumed to be well conserved and thus better able to separate the species ^34^, and a rare incidence of an overlap in wing landmarks, may indicate ongoing hybridisation^24^. Regarding the consequence of our DNA analysis, we consider that we observed a fragment of the *Culicoides* genome. Wing shape development in biting midges is probably also influenced by several genes and their expression. A previous study based on an immuno-enzymology assay showed that *C. clastrieri* and *C. festivipennis* vary by only one enzymatic character^35^ but they could not be distinguished by COI barcode in the studies of Ander et al. and Sarvašová et al. However, the authors of these studies did not cast doubt on their specific status, and Ander^24^ actually argued for possible ongoing hybridisation without any statement on their lineage divergence.

The GM technique allows species to be compared, not described. In mosquitoes, GM has been successfully applied in many studies investigating intra- and interspecific variations, parasite detection, sexual dimorphism, plasticity and deviation, separation of laboratory strains and genetic information^36^. In biting midges, the intersexual morphology specimens infested by parasites were detected by GM^28^. With respect to interspecific variation, the cryptic species seem to share approximatively 20% of the wing skeleton^26–30^. According to Sarvasova^22^, we should consider *C. clastrieri* and *C. festivipennis* as two separate species. Future research should focus on the development of nuclear markers with a higher-level phylogenetic relationship.

In conclusion, our study describes novel modelling techniques to evaluate species delineations within the *Culicoides* genus. Eight species, including two cryptic species, are clearly discriminated using selected statistical classification techniques (PLS-DA and sPLS-DA). Our study confirms *C. clastrieri* and *C. festivipennis* as two distinct species. sPLS-DA is clearly competitive in terms of computational efficiency and can separate cryptic species with fewer items than the PLS-DA classifier. We therefore propose to use combined morphological characters with a GM approach on wings, visible under a stereomiscroscope, to separate *Culicoides* species.

## Materials and methods

### Specimens and identification

Our study was conducted on 134 specimens from eight *Culicoides* species. All the specimens were collected in France using UV traps (see sampling details in Supplementary Information 1). The wings, head and abdomen (with six segments) of individual midges were mounted in Euparal solution on microscope slides for morphological identification^30^. The thorax and legs were used for DNA extraction^37^. Preliminary species identification of the specimens was based on the morphological characters and wing patterns described in the identification key of Delécolle^8^ and IIKC ^10^.

### Molecular analysis

DNA was extracted following the QIAmp_DNA Mini Kit (Qiagen, Germany) manufacturer’s recommendations as described by Augot^37^.

Polymerase chain reactions (PCR) for D1D2 and cytochrome oxidase subunit I genes were performed in a 50 µL volume using 5 µL of DNA solution and 50 pmol of primers C’1 (5′-ACCCGCTGAATTTAAGCAT-3′) and D2 (5′-TCCGTGTTTCAAGACGGG-3′) for D1D2^38^, and C1J1718 (5′-GGAGGATTTGGAAATTGATTAGT-3′) and C1N2191 (5′-CAGGTAAAATTAAAATATAAACTTCTGG- 3′) for COI^39^.

The amplification conditions for D1D2 were as follows: after an initial denaturation step at 94 °C for 3 min, followed by 35 cycles of denaturation at 94 °C for 30 s, annealing at 58 °C for 90 s, and extension at 68 °C for 60 s, then a final extension at 68 °C for 10 min. For COI, the initial denaturation step at 95 °C for 15 min, then 5 cycles at 95 °C for 40 s, 45 °C for 40 s, 72 °C for 1 min, followed by 45 cycles at 95 °C for 40 s, 50 °C for 40 s, 72 °C for 1 min and a final extension step at 72 °C for 20 min. Amplicons were analysed by electrophoresis in 1.5% agarose gel stained using the molecular weight marker 100 bp DNA Ladder (Promega) in GelGreen (Bioium).

Cleaned PCR products were sequenced by Genewiz, GmbH (www.GENEWIZ.com). PCR products were directly sequenced in both directions using the primers for DNA amplification. Sequences were corrected using the Pregap and Gap programs included in the Staden software package ^40^. Alignments and nucleotide sequence diversity among the samples were obtained using Mega v6.0. with the Kimura-2 parameter^41^.

Additionally, the COI Genbank sequences of *C. alazanicus*, *C. brunnicans*, *C. circumscriptus*, *C. clastrieri*, *C. festivipennis*, *C. furcillatus*, *C. nubeculosus* and *C. pictipennis* were also included in our molecular analyses (Supplementary Information 1).

Neighbour-joining (NJ) method (Kimura-2 parameter) analyses were performed for both markers with MEGA software version 7.0 ^42^. In order to exclude populations with the COI sequences, we divided up the species as follows: i) our specimens/species and ii) species-Genbank (Table 1). NJ trees of K2P distances were created (data not shown) to provide a graphic visualisation of clustering among different species ^43^.

### Morphological characters

The list of morphological characters used are found on the web site http://www.iikculicoides.net developed by Mathieu^10^. The raw dataset included 60 morphological characters (27 wings, 14 abdominal, 16 head and 3 leg characters) and eight species: *C. alazanicus*, *C. brunnicans*, *C. circumscriptus*, *C. clastrieri*, *C. festivipennis*, *C. furcillatus*, *C. nubeculosus* and *C. pictipennis.* For the statistical analysis, the morphological characteristics and species classification were coded as qualitative variables (see Supplementary Information 5).

### Geometric morphometric analysis

Digital images of the wings were obtained using an Olympus BX53 microscope equipped with an Olympus SC100 camera, under 10 X magnification. A set of 11 landmarks (Fig. 2) covering the wing surface was selected and recorded for each wing using the free CLIC software (https://xyom-clic.eu). Additionally, wings of species from the collection belonging to the Institut de Parasitologie et de Pathologie Tropicale de Strasbourg were included in our study (Supplementary Information 1).

### Statistical analyses

Principal component analysis (PCA) was used to explore the correlation between variables and linear discrimination analyses (LDAs) used to predict individual species based on variable values.

PCA, PLS-DA and sPLS-DA were performed using the R package mixOmics (http://www.R-project.org). PLS-DA is a supervised, multivariate modelling technique used to determine the variation within X (the morphological and landmark data), which is correlated with Y (the species). The sparse version of the technique, sPLS-DA, seeks to identify the best Kennard-Stone algorithm features that provide the best discrimination between two classes, ignoring all other features. sPLS-DA thus provides a framework for both feature selection and classification. To construct both PLS-DA and sPLS-DA, the dataset (Suppl. data) was divided into two subsets, one used for calibration (two thirds of the samples) and the other (the remaining third) used for external validation by the Kennard-Stone algorithm. The predictive ability of the final models was assessed using cross-validation. ROC analysis was used to determine optimal sensitivity and specificity to discriminate between species (with corresponding 95% confidence intervals). The area under the curve (AUC) showed the average prediction performances for the various decision thresholds. The closer the AUC is to 1, the more accurate the model^44^.

## Supplementary Information

Supplementary Information 1.

Supplementary Information 2.

Supplementary Information 3.

Supplementary Information 4.

Supplementary Information 5.

Supplementary Information 6.
