# Supplementary figures and images for: Comprehensive characterisation of Culicoides clastrieri and C. festivipennis (Diptera: Ceratopogonidae) according to morphological and morphometric characters using a multivariate approach and DNA barcode
Source: Sci Rep. 2021 Jan 13;11:521. doi: 10.1038/s41598-020-78053-3 (PMC7806617; doi:10.1038/s41598-020-78053-3)

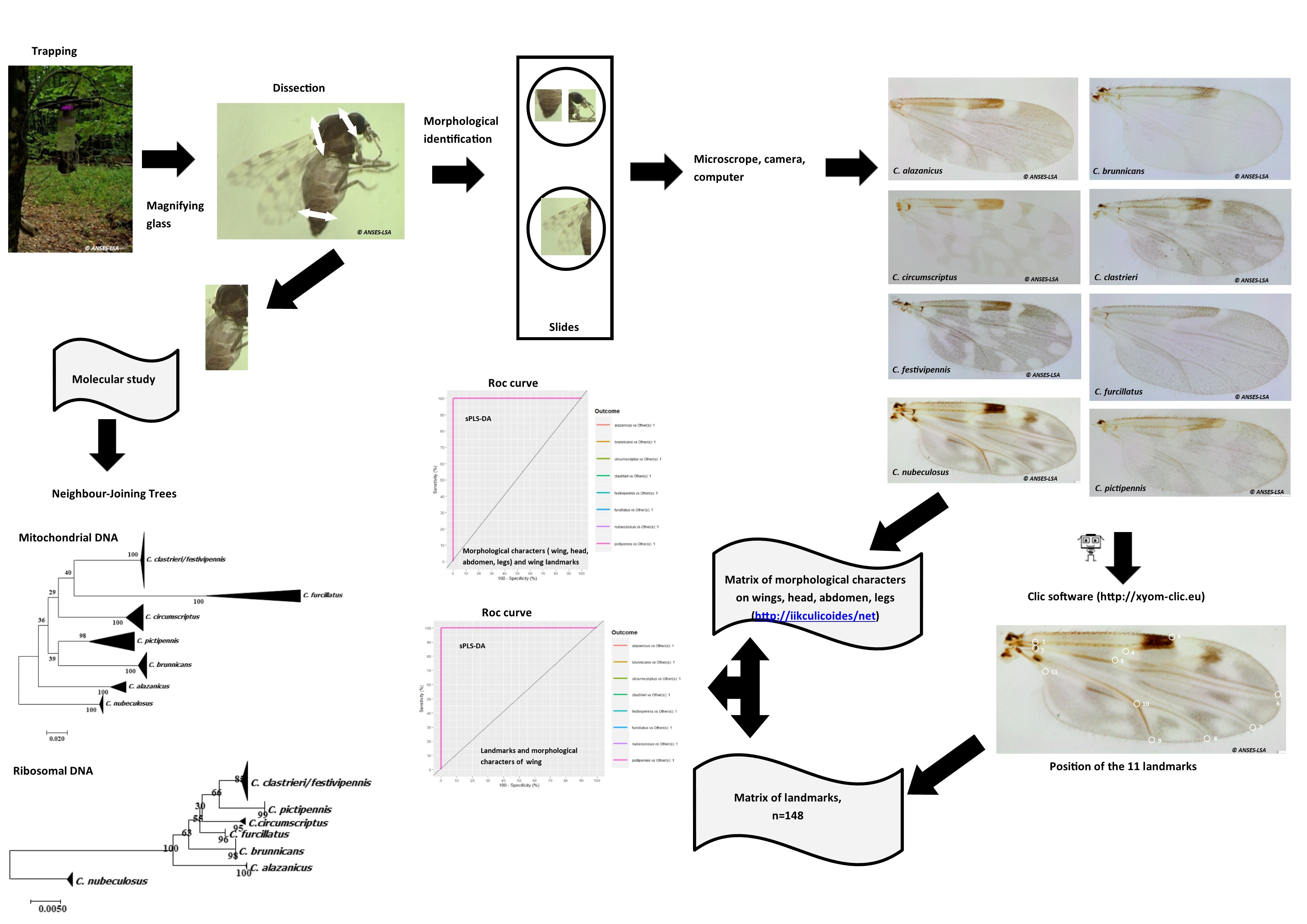

Supplement: Supplementary file 1 — Supplementary Information 1. [file 41598_2020_78053_MOESM1_ESM.jpg]
