# Supplementary material for: Comprehensive characterisation of Culicoides clastrieri and C. festivipennis (Diptera: Ceratopogonidae) according to morphological and morphometric characters using a multivariate approach and DNA barcode
Source: Sci Rep. 2021 Jan 13;11:521. doi: 10.1038/s41598-020-78053-3 (PMC7806617; doi:10.1038/s41598-020-78053-3)
Supplement: Supplementary file 2 — Supplementary Information 2. [file 41598_2020_78053_MOESM2_ESM.docx]

**Comprehensive characterisation of *Culicoides clastrieri* and *C. festivipennis* (Diptera: Ceratopogonidae) according to morphological and morphometric characters using a multivariate approach and DNA barcode**

Leila Hadj-Henni^1^, Zoubir Djerada^2^, Christine Millot^1^, Denis Augot^1*^

^1^ Usc Vecpar-ANSES LSA, EA 7510, SFR Cap Santé, Université de Reims Champagne-Ardenne, 51 rue Cognacq-Jay, 51096 Reims Cedex, France.

^2^ Department of Medical Pharmacology, EA 3801, SFR CAP Santé, Reims University Hospital, 51, rue Cognacq-Jay, 51095, Reims Cedex, France

**Email**:

(Leila Hadj-Henni) Email: leila.hadj-henni@univ-reims.fr

(Zoubir Djerada) Email: zoubir.djerada@univ-reims.fr

(Christine Millot) Email: [christine.millot@univ-reims.fr](mailto:christine.millot@univ-reims.fr)

(Denis Augot) Corresponding author should be addressed to D. A. (email: denis.augot@anses.fr)

Supplemental data-S1. *Culicoides* sampling information.

| ***Culicoides* females used in our study** | | | | | |
| --- | --- | --- | --- | --- | --- |
| **Species** | **Number** | **Coordinates** | | **Genbank accession number** | |
|  |  |  |  | **COI** | **D1D2** |
| *C. alazanicus* Dzhafarov, 1961 | 7 | 47° 43′ 26″N, 1° 43′ 55″W  46° 26′ 55″N, 1° 15′ 56″W | | MW353315, MW353316 and  MW353309- MW353313 | MW351677-MW351683 |
| *C. brunnicans* Edwards, 1939 | 23 | 49°6’4’’N, 4°7’53’’E | | KP968941– KP968963 | KP968847–KP968872 and KP968874–KP968876 |
| *C. circumscriptus* Kieffer, 1918 | 27 | [43 12′13″N, 5°46′ 38″ E](https://fr.wikipedia.org/wiki/Le_Castellet_(Var)#/maplink/1)  48° 47′ 24″N, 6° 36′ 35″E | | MW353288- MW353292, MW353293- MW353302, MW353304- MW353308 | MW351685-MW351692 |
| *C. clastrieri* Callot Kremer et Deduit, 1962 | 18 | [49°°54′16″N, 4°16′50″E](https://tools.wmflabs.org/geohack/geohack.php?pagename=Signy-le-Petit&params=49.9044_N_4.2806_E_type:city(1286)_region:FR-GES)  49° 06′ 06″N, 4° 07′ 00″E | | MW353337- MW353345 and MW353327- MW353335 | MW351649- MW351666 |
| *C. festivipennis* Kieffer, 1914 | 17 | 49° 06′ 06″N, 4° 07′ 00″E | | MW353270, MW353281, MW353290, MW353303, MW353314, MW353325, MW353336, MW353347 and MW353351- MW353359 | MW351630- MW351646 |
| *C. furcillatus* Callot, Kremer et Paradis, 1962 | 10 | 49° 31′ 18″N, 3° 04′ 17″E | | MW353326 and MW353317- MW353324 | MW351668-MW351676 |
| *C. nubeculosus* (Meigen), 1830 | 16 | 49°°06’4’’N, 4°7’53’’E | | KP969014– KP969030 | KP968924–KP968940 |
| *C. pictipennis* (Staeger), 1839 | 16 | 49° 06′ 06″N, 4° 07′ 00″E | | MW353269, MW353271- MW353280, MW353282- MW353287 | MW351680-MW351693 |
| ***Culicoides* wings added to the geometric analysis** | | | | | |
| **Species** | **Number** | **Coordinates** | | **Code** | **Remarks** |
| *C. alazanicus* | 3 | [42° 36′ 22″N, 9° 15′ 30″E](https://fr.wikipedia.org/wiki/Santo-Pietro-di-Tenda#/maplink/1) | | Col-ala-2A-France | IPPTS collection |
| *C. brunnicans* | 4 | [42° 50′ 50″N, 9° 25′ 48″E](https://fr.wikipedia.org/wiki/Pietracorbara#/maplink/1) | | Col-bru-2B-France | IPPTS collection |
| *C. clastrieri* | 3 | 48° 11′ 22″N, 7° 23′ 51″E  48° 28′ 34″N, 7° 34′ 03″E | | TYPE-cla | Type, IPPTS collection |
| *C. festivipennis* | 3 | [40° 43′ 00″N, 19° 33′ 00″E](https://fr.wikipedia.org/wiki/Fier_(Albanie)#/maplink/1) | | Col-fest-Albania | IPPTS collection |
| *C. furcillatus* | 1  3 | [41° 35′ 30″N, 9° 16′ 49″E](https://fr.wikipedia.org/wiki/Porto-Vecchio#/maplink/1) | | Type-fur  Col-fur-2A-France | Type, IPPTS collection |
| *C. nubeculosus* | 2  1 | [48° 04′ 22″N, 0° 46′ 12″W](https://fr.wikipedia.org/wiki/Laval_(Mayenne)#/maplink/1)  48° 21′ 24″N, 6° 05′ 08″E | | Col-nub-53-France  Col-nub-88-France | IPPTS collection |
| *C. pictipennis* | 4 | 42° 18′ 55″N, 9° 29′ 29″E | | Col-pic-2B-France | IPPTS collection |
| ***Culicoides* Genbank accession number added to the molecular analyses** | | | | | |
| **Species** | **Number** | | **Country** | **Remarks** |  |
| *C. alazanicus* | JQ620026-JQ620028 | | Sweden |  |  |
| *C. brunnicans* | JQ620034 | | Sweden |  |  |
| *C. circumscriptus* | JQ620054, KJ624071, HM241855, MF594386 | | Sweden, Slovakia, Spain, Turkey |  |  |
| *C. clastrieri* | JQ620061 | | Sweden |  |  |
| *C. festivipennis* | HM241860- HM241862-63  JQ620082, HQ824481 | | Spain  Sweden, Switzerland |  |  |
| *C. furcillatus* | HQ824485 | | Switzerland |  |  |
| *C. nubeculosus* | JQ620128 | | Sweden |  |  |
| *C. pictipennis* | HM241873-HM241874 | | Spain |  |  |

IPPTS: Institut de Parasitologie et Pathologie Tropicale de Strasbourg
