# Supplementary material for: Comprehensive characterisation of Culicoides clastrieri and C. festivipennis (Diptera: Ceratopogonidae) according to morphological and morphometric characters using a multivariate approach and DNA barcode
Source: Sci Rep. 2021 Jan 13;11:521. doi: 10.1038/s41598-020-78053-3 (PMC7806617; doi:10.1038/s41598-020-78053-3)
Supplement: Supplementary file 3 — Supplementary Information 3. [file 41598_2020_78053_MOESM3_ESM.docx]

**Comprehensive characterisation of *Culicoides clastrieri* and *C. festivipennis* (Diptera: Ceratopogonidae) according to morphological and morphometric characters using a multivariate approach and DNA barcode**

Leila Hadj-Henni^1^, Zoubir Djerada^2^, Christine Millot^1^, Denis Augot^1*^

^1^ Usc Vecpar-ANSES LSA, EA 7510, SFR Cap Santé, Université de Reims Champagne-Ardenne, 51 rue Cognacq-Jay, 51096 Reims Cedex, France.

^2^ Department of Medical Pharmacology, EA 3801, SFR CAP Santé, Reims University Hospital, 51, rue Cognacq-Jay, 51095, Reims Cedex, France

**Email**:

(Leila Hadj-Henni) Email: leila.hadj-henni@univ-reims.fr

(Zoubir Djerada) Email: zoubir.djerada@univ-reims.fr

(Christine Millot) Email: [christine.millot@univ-reims.fr](mailto:christine.millot@univ-reims.fr)

(Denis Augot) Corresponding author should be addressed to D. A. (email: denis.augot@anses.fr)

Supplemental-S2. The area under the curve (AUC) results obtained with PLS-DA according to models and components.

| **Model** | **Species** | **Component 1** | | **Component 2** | | **Component 3** | | **Component 4** | | **Component 5** | | **Component 6** | | **Component 7** | | **Component 8** | |
| --- | --- | --- | --- | --- | --- | --- | --- | --- | --- | --- | --- | --- | --- | --- | --- | --- | --- |
|  |  | **AUC** | ***P* value** | **AUC** | ***P* value** | **AUC** | ***P* value** | **AUC** | ***P* value** | **AUC** | ***P* value** | **AUC** | ***P* value** | **AUC** | ***P* value** | **AUC** | ***P* value** |
| **Wing landmarks** | *C. alazanicus* | 0.90 | p < 0.001 | 0.91 | p < 0.001 | 0.93 | p < 0.001 | 0.94 | p < 0.001 | 0.95 | p < 0.001 | 0.95 | p < 0.001 | 0.95 | p < 0.001 | 0.95 | p < 0.001 |
|  | *C. brunnicans* | 0.62 | 0.052 | 0.59 | 0.133 | 0.65 | 0.013 | 0.66 | p < 0.001 | 0.86 | p < 0.001 | 0.86 | p < 0.001 | 0.95 | p < 0.001 | 0.97 | p < 0.001 |
|  | *C. circumscriptus* | 0.36 | 0.019 | 0.59 | 0.137 | 0.73 | p < 0.001 | 0.96 | p < 0.001 | 0.99 | p < 0.001 | 0.99 | p < 0.001 | 0.99 | p < 0.001 | **1.00** | **p < 0.001** |
|  | *C. clastrieri* | 0.63 | 0.035 | 0.86 | p < 0.001 | 0.86 | p < 0.001 | 0.88 | p < 0.001 | 0.88 | p < 0.001 | 0.89 | p < 0.001 | 0.89 | p < 0.001 | 0.91 | p < 0.001 |
|  | *C. festivipennis* | 0.58 | 0.246 | 0.73 | p < 0.001 | 0.75 | p < 0.001 | 0.75 | p < 0.001 | 0.75 | p < 0.001 | 0.74 | p < 0.001 | 0.76 | p < 0.001 | 0.86 | p < 0.001 |
|  | *C. furcillatus* | 0.77 | < 0.001 | 0.89 | p < 0.001 | 0.88 | p < 0.001 | 0.88 | p < 0.001 | 0.88 | p < 0.001 | 0.94 | p < 0.001 | 0.94 | p < 0.001 | 0.97 | p < 0.001 |
|  | *C. nubeculosus* | 0.63 | 0.067 | 0.92 | p < 0.001 | 0.93 | p < 0.001 | 0.93 | p < 0.001 | 0.96 | p < 0.001 | **1.00** | **p< 0.001** | **1.00** | **p< 0.001** | **1.00** | **p< 0.001** |
|  | *C. pictipennis* | 0.60 | 0.149 | 0.86 | p < 0.001 | 0.86 | p < 0.001 | 0.93 | p < 0.001 | 0.98 | p < 0.001 | 0.99 | p < 0.001 | 0.99 | p < 0.001 | 0.99 | p < 0.001 |
| **Full wing (landmarks and morphological characters)** | *C. alazanicus* | 0.90 | p < 0.001 | 0.98 | p < 0.001 | 0.94 | p < 0.001 | 0.96 | p < 0.001 | 0.96 | p < 0.001 | 0.98 | p < 0.001 | 1.00 | p < 0.001 | **1.00** | **p < 0.001** |
|  | *C. brunnicans* | 0.92 | p < 0.001 | 0.99 | p < 0.001 | 0.99 | p < 0.001 | **1.00** | **p < 0.001** | **1.00** | **p < 0.001** | **1.00** | **p < 0.001** | 1.00 | p < 0.001 | **1.00** | **p < 0.001** |
|  | *C. circumscriptus* | **1.00** | **p < 0.001** | **1.00** | **p < 0.001** | **1.00** | **p < 0.001** | **1.00** | **p < 0.001** | **1.00** | **p < 0.001** | **1.00** | **p < 0.001** | 1.00 | p < 0.001 | **1.00** | **p < 0.001** |
|  | *C. clastrieri* | 0.70 | p < 0.001 | 0.90 | p < 0.001 | 0.93 | p < 0.001 | 0.92 | p < 0.001 | 0.92 | p < 0.001 | 0.97 | p < 0.001 | 1.00 | p < 0.001 | **1.00** | **p < 0.001** |
|  | *C. festivipennis* | 0.72 | p < 0.001 | 0.94 | p < 0.001 | 0.92 | p < 0.001 | 0.93 | p < 0.001 | 0.94 | p < 0.001 | **1.00** | **p < 0.001** | 1.00 | p < 0.001 | **1.00** | **p < 0.001** |
|  | *C. furcillatus* | 0.83 | p < 0.001 | 0.93 | p < 0.001 | 0.90 | p < 0.001 | 0.89 | p < 0.001 | 0.99 | p < 0.001 | 0.99 | p < 0.001 | 0.99 | p < 0.001 | 0.99 | p < 0.001 |
|  | *C. nubeculosus* | 0.60 | 0.16 | **1.00** | **p < 0.001** | 0.99 | p < 0.001 | **1.00** | **p < 0.001** | **1.00** | **p < 0.001** | **1.00** | p < 0.001 | 1.00 | p < 0.001 | **1.00** | **p < 0.001** |
|  | *C. pictipennis* | 0.52 | 0.8 | 0.62 | 0.07 | 0.73 | p < 0.001 | 0.87 | p < 0.001 | **1.00** | **p < 0.001** | **1.00** | p < 0.001 | 1.00 | p < 0.001 | **1.00** | **p < 0.001** |
| **Morphological characters (wing, head, abdomen, legs) and wing landmarks** | *C. alazanicus* | 0.91 | p < 0.001 | 0.91 | p < 0.001 | 0.96 | p < 0.001 | 0.98 | p < 0.001 | 0.98 | p < 0.001 | 0.98 | p < 0.001 | **1.00** | **p < 0.001** | **1.00** | **p < 0.001** |
|  | *C. brunnicans* | 0.90 | p < 0.001 | 0.90 | p < 0.001 | 0.99 | p < 0.001 | **1.00** | **p < 0.001** | **1.00** | **p < 0.001** | **1.00** | **p < 0.001** | **1.00** | **p < 0.001** | **1.00** | **p < 0.001** |
|  | *C. circumscriptus* | **1.00** | **p < 0.001** | **1.00** | **p < 0.001** | **1.00** | **p < 0.001** | **1.00** | **p < 0.001** | **1.00** | **p < 0.001** | **1.00** | **p < 0.001** | **1.00** | **p < 0.001** | **1.00** | **p < 0.001** |
|  | *C. clastrieri* | 0.62 | p < 0.001 | 0.72 | p < 0.001 | 0.94 | p < 0.001 | 0.94 | p < 0.001 | 0.97 | p < 0.001 | 0.95 | p < 0.001 | **0.98** | **p < 0.001** | **1.00** | **p < 0.001** |
|  | *C. festivipennis* | 0.67 | p < 0.001 | 0.82 | p < 0.001 | 0.91 | p < 0.001 | 0.91 | p < 0.001 | 0.92 | p < 0.001 | 0.98 | p < 0.001 | **0.99** | **p < 0.001** | **1.00** | **p < 0.001** |
|  | *C. furcillatus* | 0.86 | p < 0.001 | 0.84 | p < 0.001 | 0.83 | p < 0.001 | 0.92 | p < 0.001 | **1.00** | **p < 0.001** | **1.00** | **p < 0.001** | **1.00** | **p < 0.001** | **1.00** | **p < 0.001** |
|  | *C. nubeculosus* | 0.67 | 0.016 | **1.00** | **p < 0.001** | **1.00** | **p < 0.001** | **1.00** | **p < 0.001** | **1.00** | **p < 0.001** | **1.00** | **p < 0.001** | **1.00** | **p < 0.001** | **1.00** | **p < 0.001** |
|  | *C. pictipennis* | 0.60 | 0.136 | 0.39 | 0.099 | 0.62 | 0.070 | 0.67 | 0.010 | **1.00** | **p < 0.001** | **1.00** | **p < 0.001** | **1.00** | **p < 0.001** | **1.00** | **p < 0.001** |

Continued

| **Model** | **Species** | **Component 9** | | **Component 10** | | **Component 11** | | **Component 12** | | **Component 13** | | **Component 14** | | **Component 15** | | **Component 16** | |
| --- | --- | --- | --- | --- | --- | --- | --- | --- | --- | --- | --- | --- | --- | --- | --- | --- | --- |
|  |  | **AUC** | ***P* value** | **AUC** | ***P* value** | **AUC** | ***P* value** | **AUC** | ***P* value** | **AUC** | ***P* value** | **AUC** | ***P* value** | **AUC** | ***P* value** | **AUC** | ***P* value** |
| **Wing landmarks** | *C. alazanicus* | 0.95 | p < 0.001 | 0.97 | p < 0.001 | 0.97 | p < 0.001 | 0.97 | p < 0.001 | 0.97 | p < 0.001 | 0.97 | p < 0.001 | 0.97 | p < 0.001 | 0.97 | p < 0.001 |
|  | *C. brunnicans* | 0.98 | p < 0.001 | 0.98 | p < 0.001 | 0.98 | p < 0.001 | 0.98 | p < 0.001 | 0.98 | p < 0.001 | 0.98 | p < 0.001 | 0.98 | p < 0.001 | 0.98 | p < 0.001 |
|  | *C. circumscriptus* | 0.99 | p < 0.001 | 0.99 | p < 0.001 | 0.99 | p < 0.001 | 0.99 | p < 0.001 | **1.00** | **p < 0.001** | 1.00 | p < 0.001 | **1.00** | **p < 0.001** | **1.00** | **p < 0.001** |
|  | *C. clastrieri* | 0.94 | p < 0.001 | 0.95 | p < 0.001 | 0.95 | p < 0.001 | 0.96 | p < 0.001 | 0.96 | p < 0.001 | 0.97 | p < 0.001 | 0.97 | p < 0.001 | 0.97 | p < 0.001 |
|  | *C. festivipennis* | 0.86 | p < 0.001 | 0.86 | p < 0.001 | 0.86 | p < 0.001 | 0.86 | p < 0.001 | 0.87 | p < 0.001 | 0.87 | p < 0.001 | 0.90 | p < 0.001 | 0.89 | p < 0.001 |
|  | *C. furcillatus* | 0.96 | p < 0.001 | 0.96 | p < 0.001 | 0.97 | p < 0.001 | 0.97 | p < 0.001 | 0.97 | p < 0.001 | 0.97 | p < 0.001 | 0.96 | p < 0.001 | 0.97 | p < 0.001 |
|  | *C. nubeculosus* | **1.00** | **p < 0.001** | **1.00** | **p < 0.001** | **1.00** | **p < 0.001** | **1.00** | **p < 0.001** | **1.00** | **p < 0.001** | 1.00 | p < 0.001 | **1.00** | **p < 0.001** | **1.00** | **p < 0.001** |
|  | *C. pictipennis* | 0.99 | p < 0.001 | **1.00** | **p < 0.001** | **1.00** | **p < 0.001** | **1.00** | **p < 0.001** | **1.00** | **p < 0.001** | 1.00 | p < 0.001 | **1.00** | **p < 0.001** | **1.00** | **p < 0.001** |
| **Full wing (landmarks and morphological characters)** | *C. alazanicus* | **1.00** | **p < 0.001** | **1.00** | **p < 0.001** | **1.00** | **p < 0.001** | **1.00** | **p < 0.001** | **1.00** | **p < 0.001** | **1.00** | **p < 0.001** | **1.00** | **p < 0.001** | **1.00** | **p < 0.001** |
|  | *C. brunnicans* | **1.00** | **p < 0.001** | **1.00** | **p < 0.001** | **1.00** | **p < 0.001** | **1.00** | **p < 0.001** | **1.00** | **p < 0.001** | **1.00** | **p < 0.001** | **1.00** | **p < 0.001** | **1.00** | **p < 0.001** |
|  | *C. circumscriptus* | **1.00** | **p < 0.001** | **1.00** | **p < 0.001** | **1.00** | **p < 0.001** | **1.00** | **p < 0.001** | **1.00** | **p < 0.001** | **1.00** | **p < 0.001** | **1.00** | **p < 0.001** | **1.00** | **p < 0.001** |
|  | *C. clastrieri* | **1.00** | **p < 0.001** | **1.00** | **p < 0.001** | **1.00** | **p < 0.001** | **1.00** | **p < 0.001** | **1.00** | **p < 0.001** | **1.00** | **p < 0.001** | **1.00** | **p < 0.001** | **1.00** | **p < 0.001** |
|  | *C. festivipennis* | **1.00** | **p < 0.001** | **1.00** | **p < 0.001** | **1.00** | **p < 0.001** | **1.00** | **p < 0.001** | **1.00** | **p < 0.001** | **1.00** | **p < 0.001** | **1.00** | **p < 0.001** | **1.00** | **p < 0.001** |
|  | *C. furcillatus* | **1.00** | **p < 0.001** | **1.00** | **p < 0.001** | **1.00** | **p < 0.001** | **1.00** | **p < 0.001** | **1.00** | **p < 0.001** | **1.00** | **p < 0.001** | **1.00** | **p < 0.001** | **1.00** | **p < 0.001** |
|  | *C. nubeculosus* | **1.00** | **p < 0.001** | **1.00** | **p < 0.001** | **1.00** | **p < 0.001** | **1.00** | **p < 0.001** | **1.00** | **p < 0.001** | **1.00** | **p < 0.001** | **1.00** | **p < 0.001** | **1.00** | **p < 0.001** |
|  | *C. pictipennis* | **1.00** | **p < 0.001** | **1.00** | **p < 0.001** | **1.00** | **p < 0.001** | **1.00** | **p < 0.001** | **1.00** | **p < 0.001** | **1.00** | **p < 0.001** | **1.00** | **p < 0.001** | **1.00** | **p < 0.001** |
| **Morphological characters (wing, head, abdomen, legs) and wing landmarks** | *C. alazanicus* | **1.00** | **p < 0.001** | **1.00** | **p < 0.001** | **1.00** | **p < 0.001** | **1.00** | **p < 0.001** | **1.00** | **p < 0.001** | **1.00** | **p < 0.001** | **1.00** | **p < 0.001** | **1.00** | **p < 0.001** |
|  | *C. brunnicans* | **1.00** | **p < 0.001** | **1.00** | **p < 0.001** | **1.00** | **p < 0.001** | **1.00** | **p < 0.001** | **1.00** | **p < 0.001** | **1.00** | **p < 0.001** | **1.00** | **p < 0.001** | **1.00** | **p < 0.001** |
|  | *C. circumscriptus* | **1.00** | **p < 0.001** | **1.00** | **p < 0.001** | **1.00** | **p < 0.001** | **1.00** | **p < 0.001** | **1.00** | **p < 0.001** | **1.00** | **p < 0.001** | **1.00** | **p < 0.001** | **1.00** | **p < 0.001** |
|  | *C. clastrieri* | **1.00** | **p < 0.001** | **1.00** | **p < 0.001** | **1.00** | **p < 0.001** | **1.00** | **p < 0.001** | **1.00** | **p < 0.001** | **1.00** | **p < 0.001** | **1.00** | **p < 0.001** | **1.00** | **p < 0.001** |
|  | *C. festivipennis* | **1.00** | **p < 0.001** | **1.00** | **p < 0.001** | **1.00** | **p < 0.001** | **1.00** | **p < 0.001** | **1.00** | **p < 0.001** | **1.00** | **p < 0.001** | **1.00** | **p < 0.001** | **1.00** | **p < 0.001** |
|  | *C. furcillatus* | **1.00** | **p < 0.001** | **1.00** | **p < 0.001** | **1.00** | **p < 0.001** | **1.00** | **p < 0.001** | **1.00** | **p < 0.001** | **1.00** | **p < 0.001** | **1.00** | **p < 0.001** | **1.00** | **p < 0.001** |
|  | *C. nubeculosus* | **1.00** | **p < 0.001** | **1.00** | **p < 0.001** | **1.00** | **p < 0.001** | **1.00** | **p < 0.001** | **1.00** | **p < 0.001** | **1.00** | **p < 0.001** | **1.00** | **p < 0.001** | **1.00** | **p < 0.001** |
|  | *C. pictipennis* | **1.00** | **p < 0.001** | **1.00** | **p < 0.001** | **1.00** | **p < 0.001** | **1.00** | **p < 0.001** | **1.00** | **p < 0.001** | **1.00** | **p < 0.001** | **1.00** | **p < 0.001** | **1.00** | **p < 0.001** |

Continued

| **Model** | **Species** | **Component 17** | | **Component 18** | | **Component 19** | | **Component 20** | |
| --- | --- | --- | --- | --- | --- | --- | --- | --- | --- |
|  |  | **AUC** | ***P* value** | **AUC** | ***P* value** | **AUC** | ***P* value** | **AUC** | ***P* value** |
| **Wing landmarks** | *C. alazanicus* | 0.97 | p < 0.001 | 0.97 | p < 0.001 | 0.97 | p < 0.001 | 0.98 | p < 0.001 |
|  | *C. brunnicans* | 0.99 | p < 0.001 | 0.99 | p < 0.001 | 0.99 | p < 0.001 | 0.99 | p < 0.001 |
|  | *C. circumscriptus* | **1.00** | **p < 0.001** | **1.00** | **p < 0.001** | **1.00** | **p < 0.001** | **1.00** | **p < 0.001** |
|  | *C. clastrieri* | 0.97 | p < 0.001 | 0.97 | p < 0.001 | 0.97 | p < 0.001 | 0.98 | p < 0.001 |
|  | *C. festivipennis* | 0.90 | p < 0.001 | 0.90 | p < 0.001 | 0.91 | p < 0.001 | 0.91 | p < 0.001 |
|  | *C. furcillatus* | 0.98 | p < 0.001 | 0.98 | p < 0.001 | 0.98 | p < 0.001 | 0.98 | p < 0.001 |
|  | *C. nubeculosus* | **1.00** | **p < 0.001** | **1.00** | **p < 0.001** | **1.00** | **p < 0.001** | **1.00** | **p < 0.001** |
|  | *C. pictipennis* | **1.00** | **p < 0.001** | **1.00** | **p < 0.001** | **1.00** | **p < 0.001** | **1.00** | **p < 0.001** |
| **Full wing (landmarks and morphological characters)** | *C. alazanicus* | **1.00** | **p < 0.001** | **1.00** | **p < 0.001** | **1.00** | **p < 0.001** | **1.00** | **p < 0.001** |
|  | *C. brunnicans* | **1.00** | **p < 0.001** | **1.00** | **p < 0.001** | **1.00** | **p < 0.001** | **1.00** | **p < 0.001** |
|  | *C. circumscriptus* | **1.00** | **p < 0.001** | **1.00** | **p < 0.001** | **1.00** | **p < 0.001** | **1.00** | **p < 0.001** |
|  | *C. clastrieri* | **1.00** | **p < 0.001** | **1.00** | **p < 0.001** | **1.00** | **p < 0.001** | **1.00** | **p < 0.001** |
|  | *C. festivipennis* | **1.00** | **p < 0.001** | **1.00** | **p < 0.001** | **1.00** | **p < 0.001** | **1.00** | **p < 0.001** |
|  | *C. furcillatus* | **1.00** | **p < 0.001** | **1.00** | **p < 0.001** | **1.00** | **p < 0.001** | **1.00** | **p < 0.001** |
|  | *C. nubeculosus* | **1.00** | **p < 0.001** | **1.00** | **p < 0.001** | **1.00** | **p < 0.001** | **1.00** | **p < 0.001** |
|  | *C. pictipennis* | **1.00** | **p < 0.001** | **1.00** | **p < 0.001** | **1.00** | **p < 0.001** | **1.00** | **p < 0.001** |
| **Morphological characters (wing, head, abdomen, legs) and wing landmarks** | *C. alazanicus* | **1.00** | **p < 0.001** | **1.00** | **p < 0.001** | **1.00** | **p < 0.001** | **1.00** | **p < 0.001** |
|  | *C. brunnicans* | **1.00** | **p < 0.001** | **1.00** | **p < 0.001** | **1.00** | **p < 0.001** | **1.00** | **p < 0.001** |
|  | *C. circumscriptus* | **1.00** | **p < 0.001** | **1.00** | **p < 0.001** | **1.00** | **p < 0.001** | **1.00** | **p < 0.001** |
|  | *C. clastrieri* | **1.00** | **p < 0.001** | **1.00** | **p < 0.001** | **1.00** | **p < 0.001** | **1.00** | **p < 0.001** |
|  | *C. festivipennis* | **1.00** | **p < 0.001** | **1.00** | **p < 0.001** | **1.00** | **p < 0.001** | **1.00** | **p < 0.001** |
|  | *C. furcillatus* | **1.00** | **p < 0.001** | **1.00** | **p < 0.001** | **1.00** | **p < 0.001** | **1.00** | **p < 0.001** |
|  | *C. nubeculosus* | **1.00** | **p < 0.001** | **1.00** | **p < 0.001** | **1.00** | **p < 0.001** | **1.00** | **p < 0.001** |
|  | *C. pictipennis* | **1.00** | **p < 0.001** | **1.00** | **p < 0.001** | **1.00** | **p < 0.001** | **1.00** | **p < 0.001** |
